# Novel Avenues for Plant Protection: Plant Propagation by Somatic Embryogenesis Enhances Resistance to Insect Feeding

**DOI:** 10.3389/fpls.2018.01553

**Published:** 2018-10-26

**Authors:** Adriana Puentes, Karl-Anders Högberg, Niklas Björklund, Göran Nordlander

**Affiliations:** ^1^Department of Ecology, Swedish University of Agricultural Sciences (SLU), Uppsala, Sweden; ^2^Skogforsk, The Forestry Research Institute of Sweden, Svalöv, Sweden

**Keywords:** emblings, herbivore damage, genetic variation, *Hylobius abietis*, *Picea abies*, plant biotic defense, plant propagation, somatic embryogenesis

## Abstract

Somatic embryogenesis (SE), a clonal propagation method utilizing somatic cells, occurs under conditions that activate plant stress adaptation mechanisms such as production of protective secondary metabolites. Surprisingly, possible differences in susceptibility to insect pests between SE-generated and conventionally cultivated plants have not been previously explored. Here, we recorded frequencies and levels of bark-feeding damage by pine weevils (*Hylobius abietis*) in two large field trials, consisting of emblings (SE-propagated plants) and seedlings from 50 half-sib Norway spruce (*Picea abies*) families. We found that emblings were less frequently attacked by pine weevils, and when attacked, they were damaged to a lesser extent than seedlings. Moreover, we detected significant additive genetic variation in damage levels received by plants, indicating a heritable component to differences in resistance to insect herbivory among half-sib families. We present first-time evidence that emblings can be more resistant than seedlings to herbivorous insect damage, thus, SE appears to confer a previously unknown plant protection advantage. This finding indicates novel avenues to explore mechanisms underlying plant resistance and new approaches to develop non-toxic measures against insect pests.

## Introduction

Somatic embryogenesis (SE), a clonal propagation process in which embryos are derived from somatic (non-sexual) cells, has great potential for exploiting genetic gains obtained in breeding through very rapid propagation of superior genotypes. Studies of SE-generated conifer plants (hereafter emblings) have shown that such plants are similar to seedlings in terms of growth and morphological traits, even in field trials up to 7 years after planting (e.g., Grossnickle and Major, 1994; O’Neill et al., 2005). However, little is known about emblings’ properties apart from their appearance and growth attributes relevant to production in forestry. This is despite SE’s known potential to generate genetic, epigenetic or phenotypic variation in propagated plant material (Etienne et al., 2016). As SE involves reprogramming of gene expression patterns leading to changes in physiology and metabolism of cultured cells (Namasivayam, 2007), the process could affect traits of emblings that are not morphologically evident. Moreover, morphological or other changes could profoundly affect interactions of SE-propagated plants with their abiotic and biotic environment, either directly or indirectly. Thus, the changes induced by SE, the mechanisms involved, and their practical implications all clearly warrant close attention.

Briefly, somatic embryos are exposed to artificial *in vitro* environments, while zygotic embryos develop in an ovule-enclosed environment, so there are stark differences in both physical factors (e.g., light quality, photoperiod, and temperatures) and chemical factors (e.g., levels of plant growth regulators). Somatic embryos are also exposed to large exogenous applications of the plant hormone abscisic acid (ABA) to induce their maturation. Consequently, internal ABA concentrations are often orders of magnitude higher in somatic embryos than in zygotic embryos, and the difference is especially pronounced in conifers (von Aderkas et al., 2001). Moreover, the artificial environment induces stress adaptation responses in the developing embryos, resulting (*inter alia*) in increased production of protective secondary metabolites (von Aderkas et al., 2015; Klimaszewska et al., 2016). For example, strong upregulation of several genes involved in the biosynthesis of secondary metabolites including flavonols has been detected at the onset of SE maturation in *Pinus pinaster* (Morel et al., 2014) and *Theobroma cacao* (Maximova et al., 2014). More generally, plants’ physiological, metabolic and chemical properties may be changed by alterations in the expression of both developmental and stress-related genes induced by the tissue culture conditions associated with SE (Neelakandan and Wang, 2012; Kumar and Van Staden, 2017).

The documented responses involving secondary compound production during the SE process suggest that interactions of SE-plants with biotic stresses could be affected. For instance, studies of host-pathogen interactions in early tissue development have shown that fungal mycelial growth is strongly reduced by inhibitory substances released by somatic embryos when grown together in culture (Terho et al., 2000; Vookova et al., 2006; Hrib et al., 2013; Nawrot-Chorabik et al., 2016). Furthermore, changes in secondary compounds early in development may be persistent, as shown by plants propagated by SE to obtain compounds of medicinal interest. For instance, levels of secondary metabolites in the forest trees *Magnolia dealbata* and *Nothapodytes foetida* reportedly remained elevated in emblings after growth for 6 months in the greenhouse and 2 years in the field, respectively (Fulzele and Satdive, 2003; Domínguez et al., 2010). Secondary compounds also play important roles in plant-insect herbivore interactions by deterring, reducing or even stopping feeding by herbivores (Agrawal and Weber, 2015). Consequently, SE-mediated changes in secondary metabolite production, and/or other traits, could affect plants’ future susceptibility, and the potential utility of exploiting SE to improve plants’ resistance to insect herbivores should be examined.

In the study presented here, we recorded frequencies and levels of pine weevil (*Hylobius abietis*) damage in field trials with Norway spruce (*Picea abies*) plants in southern Sweden. The trials consisted of both emblings and seedlings from 50 half-sib families from breeding populations generated and maintained by Skogforsk (the Forestry Research Institute of Sweden). To our knowledge (based on an extensive literature search, see Supplementary Information) no study has previously examined how emblings and seedlings differ in their ability to resist (limit or reduce) herbivore damage. Our results provide first-time evidence that SE-plants have an advantage over conventionally cultivated plants as they are less frequently attacked, and even when attacked, they receive less damage by a bark-feeding herbivore.

## Materials and Methods

### Plant-Herbivore Study System

The pine weevil *Hylobius abietis* (L.) (Coleoptera: Curculionidae) is widely distributed in Europe and Asia, and is associated with many species of the conifer tree family Pinaceae for both breeding and adult feeding (Day et al., 2004; Wallertz et al., 2014). The adult weevils consume phloem tissue of thin bark and frequently kill young tree plants by girdling the stem (Wainhouse, 2004). Damage by pine weevils to planted conifer seedlings is a main threat to successful forest regeneration in large parts of Europe (Långström and Day, 2004; Nilsson et al., 2010). Measures to counter this damage, including direct protection of plants with insecticides or protective coatings and various risk-reducing actions, are usually essential (Nordlander et al., 2011; Willoughby et al., 2017). Complementary strategies to boost plant resistance and survival rates are currently being investigated, including the induction of plant defenses with the plant hormone methyl jasmonate (Zas et al., 2014; Fedderwitz et al., 2016) and enhancing genetically based resistance by selecting parents with low susceptibility from breeding populations (Zas et al., 2017).

### Plant Material

The plant material examined consisted of 50 Norway spruce (*Picea abies* (L.) Karst.) half-sib families, obtained by harvesting seeds from plus trees growing in a clonal archive close to Skogforsk’s research station Ekebo in southernmost Sweden. Immature seeds collected at the end of July 2011 were used to initiate somatic embryo cultures and mature seeds from the same trees were collected in October 2011 for seedling production. These seeds were sown in 150 ml containers and plants remained in them for the 1st year’s growth.

The SE-based propagation started with the initiation of cell lines from 40 embryos per family. This was done following Högberg et al. (1998), with an additional step before acclimation to *ex vitro* conditions: placement of germinants in vessels that allowed root development in liquid nutrient solution and shoot growth in air (Högberg et al., 2001). There were substantial losses of cell lines during propagation and the distribution of cell lines (clones) among families was uneven, which are typical features of SE propagation (Högberg, 2012). However, to achieve evenness among the three field trials, the plant material was later divided into three groups which contained a similar number of plants (Table 1).

**Table 1:** Description of sites for the experimental field trials, and numbers of half-sib families, within-family and clone replication for Norway spruce (*Picea abies*) seedlings and emblings at each site.

	Trial A	Trial B	Trial C
Locality name	Toresbo	Åsmundsryd	Remningstorp
Latitude	56° 41′	56° 50′	58° 27′
Longitude	15° 47′	16° 02′	13° 39′
h.a.s.l.	120 m	80 m	135 m
Previous land use	Forest	Forest	Farm land
Clear-cut year	2015	2014	N/A
Soil texture	Sandy-silty till	Sandy-silty till	Sand with gravel
Site index^∗^	G33	G31	G31
Spacing among plants	2.25 m × 1.4 m	2.25 m × 1.8 m	1.5 m × 1.4 m
No. of families	50	50	50
No. of unpruned seedlings	133	141	150
No. of pruned seedlings	196	193	184
No. of emblings	954	959	958
No. of emblings/family	1–9	1–8	1–7
No. of emblings/SE clone	1–7	1–7	1–6


*^∗^Expressed according to a Swedish system (Hägglund and Lundmark, 1987).*

Both seedlings and emblings were transplanted to 0.8 l containers in May 2014 and allowed to grow until December 2014, when they were transferred to freezing storage at -4°C. At that time, for purposes not related to this study, all emblings and 12 seedlings per family were pruned to collect cuttings (4–12 from each embling, mean number of cuttings = 11.4; and 12 cuttings from each seedling). The mean height and SE of pruned seedlings and emblings at the time of cutting collection were 66.5 ± 14.5 cm and 52.1 ± 14.7 cm, respectively.

### Field Trials

In the spring of 2015, three field trials were established. Trials A and B were established on forest land (scarified by disc-trenching before planting) where high levels of pine weevil damage (PWD) were expected. A comparison trial, designated Trial C, was established on abandoned farm land and no PWD was expected here as there were no conifer stumps emitting host odors that attract pine weevils to the site (Table 1). The trials were included in a project that was initially intended to study effects of SE on plant establishment and growth, but it also provided a fortuitous opportunity to compare emblings’ and seedlings’ susceptibility to insect damage. An incomplete randomized block design was applied, allowing replications within blocks due to the unbalanced outcome of the SE propagation. Numbers of replications of clones and families within blocks approximately matched the proportions obtained after propagation. Furthermore, two types of seedlings (pruned and unpruned) and emblings (all pruned) were intermixed to avoid spatial aggregation of any plant type.

Pine weevil damage was assessed in plants included in the two trials on clear-cut forest sites, A and B (Table 1). As expected, there was no PWD in Trial C. As a protective measure against damage by pine weevils during the 1st year, all seedlings and emblings in Trials A and B were individually treated with insecticide (Merit Forest WG, active substance imidacloprid; Bayer AB) in both spring and autumn 2015, but no insecticide treatment was applied in 2016. Because the plants were considerably larger than those normally used in Swedish plantations, they were expected to survive even very high levels of PWD.

### Damage and Growth Assessment

After 2 years of growth in the field (autumn 2016), PWD was assessed in Trials A and B, and plant height was measured in all trials. Since the extent of damage differed between Trials A and B, damage to plants included in them was assessed in different ways. In Trial A, damage was very extensive and nearly all plants were attacked, allowing us to assess PWD in terms of six classes based on the percentage of total stem area debarked (Table 2). In Trial B, there was less damage (more than half of the plants were not attacked), which allowed us to assess the risk of plants being attacked. In this trial, PWD was assessed by estimating the amount of stem area debarked (cm^2^) by pine weevils per plant.

**Table 2:** Classes of pine weevil damage (PWD) used to record damage levels for all plants in trial A.

PWD class	Damage level (%)	PWD mid-class
0	0	0
1	>0 to ≤1	0.5%
2	>1 to ≤5	3%
3	>5 to ≤30	17.5%
4	>30 to ≤50	40%
5	>50	60%^∗^


*Extent of damage in each class is expressed as a range of minimum and maximum percentages of debarked stem area. Mid-class values (PWD mid-class) were used to express PWD as a continuous variable. ^∗^Maximum PWD was 70% which means a mid-class value of 60%.*

### Statistical Analyses

#### PWD and Plant Growth

To assess the risk of plants of being attacked by pine weevils (Trial B), we expressed the incidence of pine weevil attack as a binary trait (0 or 1) then determined the significance of differences between emblings and seedlings using a χ^2^-test. To assess effects on the extent of damage in Trial B, debarked area was used as the response variable. In Trial A, the binary approach would not have been informative, as almost all plants had been attacked, so the percentages of stem area debarked, expressed as five classes or mid-class values, was used as the response variable (Table 2). Effects on plant height and PWD were examined using the following mixed model:

(1) y=m+b+t+f+c(f)+e

Here, *y* = observed values, *m* = mean, *b* = fixed effect of block, *t* = fixed effect of plant type (i.e., emblings, pruned, and unpruned seedlings), *f* = random effect of family, *c(f)* = random effect of clone within family, and *e* = random error term. Estimates of effects were obtained by solving the mixed model equation (Henderson, 1984; Quaas, 1988) using SAS software (SAS Institute Inc, 2008), and the significance of differences among fixed effects was evaluated by *F*-tests. For trial A, a χ^2^-test was also conducted to examine differences among fixed effects, as the frequency of individuals in the different PWD classes deviated somewhat from a normal distribution.

#### Genetic Variation in Resistance Against PWD

To explore the genetic contribution to variation among half-sib families in resistance to PWD, we fitted the following model:

(2) $$y=X_{1}b+X_{2}c+Za+e$$

Here, *y* = observed value, *b* = a vector of fixed effects of block, *c* = a vector of fixed effects of plant type (i.e., emblings, pruned, and unpruned seedlings), *a* = a vector of random effects of genotype, *X*_1_ = a design matrix allocating observations to block, *X*_2_ = a design matrix allocating observations to plant type, *Z* = a relationship matrix allocating observations to genotype and *e* = a vector of random errors. The genetic model was further expanded to reflect microsite variations affecting growth and weevil damage by including the following first-order auto-regressive correlation matrix partitioning error variance:

(3) $$R=\sigma_{\xi }^{2}[\mathrm{AR}(\rho_{row})⊗\mathrm{AR}(\rho_{col})]+\sigma_{\eta }^{2}I$$

Here, *R* = residual, $\sigma_{\xi }^{2}$ = spatial residual variance, $\sigma_{\eta }^{2}$ = independent residual variance, *I* = an identity matrix, AR(ρ_row_) = an auto-regression correlation matrix in row direction, AR(ρ_col_) = an auto-regression correlation matrix in column direction and ⊗ denotes direct product.

The phenotypic variance $\left(\sigma_{p}^{2}\right)$ and narrow-sense heritabilities (*h*^2^) were estimated as:

$$\sigma_{p}^{2}=\sigma_{a}^{2}+\sigma_{e}^{2}\;\;\;\;\;h^{2}=\frac{\sigma_{a}^{2}}{\sigma_{p}^{2}}$$

Here, $\sigma_{a}^{2}$ is the additive genetic variance and $\sigma_{e}^{2}$ is the residual variance.

To estimate genetic correlations between plant height and PWD, variances and covariances between traits were estimated by bivariate analysis, using ASReml (Gilmour et al., 2009). The bivariate model was an expansion of the univariate model that included a vector of values for the two traits, and matrices corresponding to the fixed and random effect design for each trait. Variances and covariances of the random effects were modeled as:

(4) $$\mathrm{Var}\left[\begin{array}{c} a\\ e \end{array}\right]=\left[\begin{array}{cc} G⊗A & 0\\ 0 & R⊗I \end{array}\right]$$

Here, ***a*** is the random additive vector and ***e*** is the error vector, ***G*** is the additive genetic variance/covariance matrix, ***A*** is the numerator relationship matrix, ***R*** is the residual variance/covariance matrix and ***I*** is an identity matrix. The symbol ⊗ denotes direct matrix product.

Additive genetic correlations between traits were calculated as:

$$r_{a12}=\sigma_{a12}/(\sigma_{a1}\sigma_{a2})$$

Here, *r_a_*_12_ is the additive genetic correlation between traits 1 and 2, σ*_a_*_12_ is the additive covariance between traits 1 and 2, σ*_a_*_1_ and σ*_a_*_2_ are the square roots of the additive variance for traits 1 and 2, respectively.

## Results

### PWD and Plant Growth

Damage by bark-feeding pine weevils is presented for two (Trials A and B) of three large field trials (Table 1), as the third field trial (Trial C) was a comparison trial lacking pine weevil infestation. Plant mortality was low in all trials (proportion of dead plants: 4.5, 0.4, and 0.9% in Trials A, B, and C, respectively) by the end of the second field season. For purposes not related to this study, all emblings and a sub-set of seedlings had been pruned to collect cuttings prior to planting (see section ‘Plant Material’ in Materials and Methods). Thus, the results that follow include comparison of emblings to both kinds of seedlings (pruned and unpruned).

**FIGURE 1:**
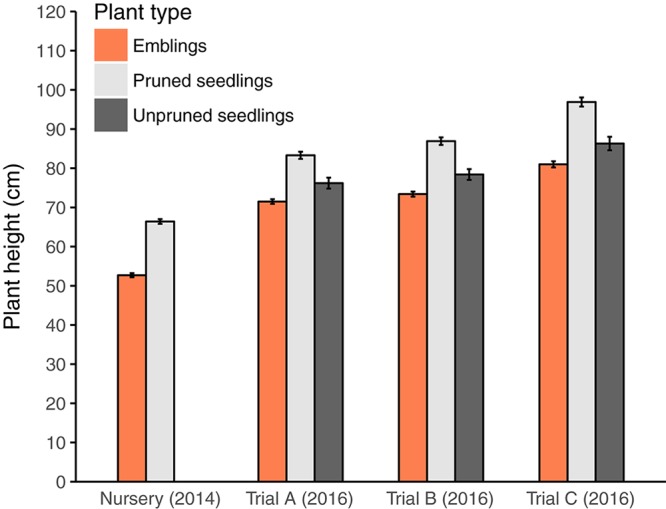
Mean plant height (±SE) after plant production in the nursery (2014) but before seedlings were pruned, and after 2 years of growth in the field experimental trials (Trials A, B, and C) in 2016. Note that in trial C (on abandoned farmland) plants did not receive any pine weevil damage.

The average heights of the plants, by the end of the second field season, were very similar among trials (mean ± SE, 73.9 ± 0.37 and 75.7 ± 0.37 cm in Trials A and B, respectively). The average height of plants at the farm land site in Trial C was 84.7 ± 0.44 cm. Even in trial C, where plants received no PWD, emblings had similar growth rates to seedlings; they were slightly shorter initially and remained slightly shorter throughout the experimental period (Figure 1). Pruned seedlings were significantly taller than unpruned seedlings, but they were probably already taller in the nursery, due to unintentional bias toward selecting larger seedlings for cutting collection.

**FIGURE 2:**
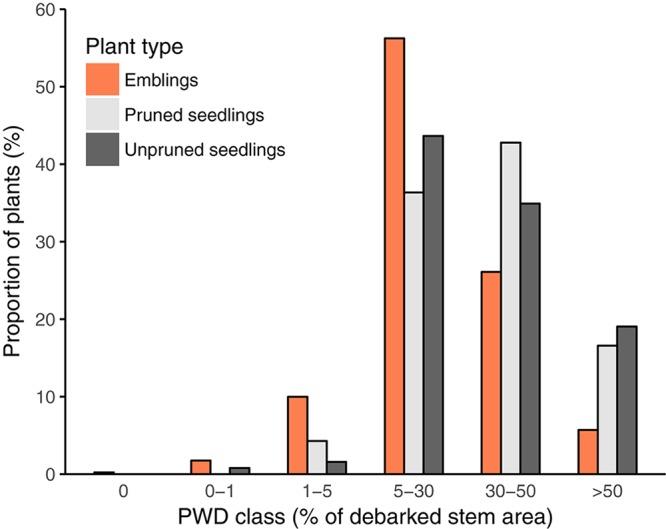
Proportions of plants in each of the six Pine Weevil Damage classes (PWD class, Table 2) used to record levels of damage for all plants (emblings, pruned, and unpruned seedlings) in trial A. Extent of damage in each class is expressed as a range of minimum and maximum values of percentage of debarked stem area.

With respect to PWD, all plants except two emblings were attacked in Trial A, while 41% of plants were attacked in Trial B. Given that damage was extensive and nearly all plants were attacked in Trial A, we report damage in terms of six damage classes (% debarked stem area, Table 2); for Trial B, we report the risk of attack and stem area debarked (cm^2^). In trial A, emblings were significantly less damaged than pruned and unpruned seedlings according to a χ^2^-test using an adjusted scale (Figure 2; χ^2^ = 85.86, *df* = 8, *p* < 0.0001; damage classes 0 and 1 were merged prior to analysis as the frequency in class 0 was very low). In trial B, the proportion of emblings attacked was significantly lower (39%) than the proportions of both pruned and unpruned seedlings (47 and 51%, respectively) (χ^2^= 10.64, *df* = 2, *p* = 0.0049). Moreover, the proportion of stem area debarked was also lower for emblings compared to seedlings in this trial (mean proportion of stem area debarked ± SE, emblings 0.22 ± 0.02, unpruned seedlings 0.30 ± 0.05, pruned seedlings 0.40 ± 0.04; Table 5).

**FIGURE 3:**
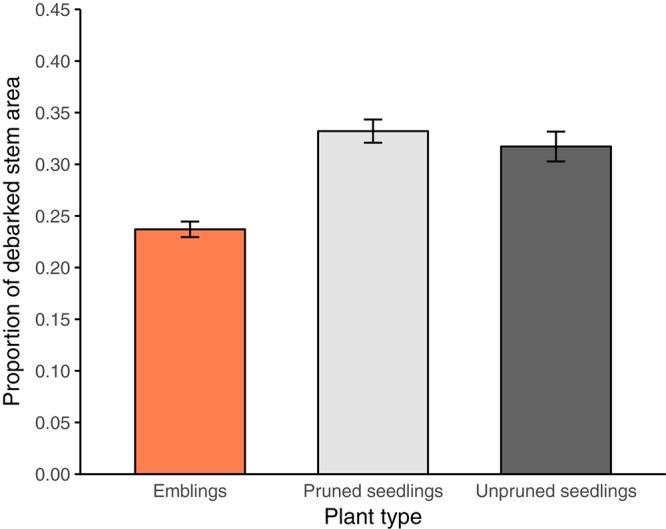
Estimated mean proportion of debarked stem area (±SE) for each plant type (emblings, pruned, and unpruned seedlings) in trial A. Estimates represent averages of mid-class values across pine weevil damage (PWD) classes (Table 2) for each plant type.

Variance analysis provided further evidence that emblings were less damaged than seedlings (Figure 3 and Tables 3, 4), as the effect of plant type (emblings, pruned, or unpruned seedlings) was also significant when damage was assumed to follow a normal distribution. Use of mid-class damage values in the analysis of PWD in Trial A did not change this result (Table 3). Family effects (50 half-sib Norway spruce families) explained less than 5% of the total random variance in height in both trials, while clone effects (SE-propagated cell lines) within family explained considerably more (Tables 3, 4). Corresponding values for PWD in Trial A showed similar contributions of family and clone to random variance (Table 3), while family effects in Trial B explained clearly less variance than clone effects (Table 4).

**Table 3:** Results of Analysis of Variance (ANOVA) using mixed models to examine differences in pine weevil damage (PWD; in classes 0–5, or as a continuous variable based on mid-class values, Table 2) between Norway spruce emblings and two types of seedlings in Trial A.

	PWD classes (0–5)			PWD mid-class value			Plant height (cm)		
			
Source of variation	*df*	*F*	*p*	*df*	*F*	*p*	*df*	*F*	*p*
**Fixed effects**									
Block	4,607	74.1	**<0.0001**	4,607	75.9	**<0.0001**	4,607	27.4	**<0.0001**
Plant type	2,607	41.2	**<0.0001**	2,607	44.3	**<0.0001**	2,607	71.1	**<0.0001**

**Random effects**	**Var. expl.**	***Z*-value**	***p***	**Var. expl.**	***Z*-value**	***p***	**Var. expl.**	***Z*-value**	***p***

Family	6.1%	2.4	**0.009**	7.3%	2.6	**0.005**	3.6%	1.4	0.076
Clone	10.8%	3.0	**0.001**	8.4%	2.4	**0.008**	30.3%	6.7	**<0.0001**


*Models included block and plant type (emblings, unpruned seedlings, and pruned seedlings) as fixed factors, and half-sib family and clone (within family) as random factors. *Var. expl.* for random effects indicates the percentage of total random variance (Family, Clone, and Residual) explained by each component.*

**Table 4:** Results of Analysis of Variance (ANOVA) using mixed models to examine differences in levels of pine weevil damage (PWD; debarked stem area cm^2^) between Norway spruce emblings and two types of seedlings in Trial B.

	PWD (cm^2^)			Plant height (cm)		
		
Source of variation	*df*	*F*	*p*	*df*	*F*	*p*
**Fixed effects**						
Block	3,629	13.2	**<0.0001**	3,629	14.8	**<0.0001**
Plant type	2,629	10.5	**<0.0001**	2,629	86.2	**<0.0001**

**Random effects**	**Var. expl.**	***Z*-value**	***p***	**Var. expl.**	***Z*-value**	***p***

Family	1.0%	1.0	0.153	4.6%	2.0	**0.024**
Clone	8.2%	2.1	**0.019**	25.9%	6.4	**<0.0001**


*Models included block and plant type (emblings, unpruned seedlings, and pruned seedlings) as fixed factors, and half-sib family and clone (within family) as random factors. *Var. expl.* for random effects indicates the percentage of total random variance (Family, Clone, and Residual) explained by each component.*

### Genetic Variation in Resistance to PWD

Our results show that there is a genetic component to variation among half-sib families in plant growth and resistance to PWD. We found significant additive genetic variation for plant height and levels of insect damage received by plants. The heritability estimates for levels of PWD differed between trials (*h*^2^ = 0.31 and 0.10 for trials A and B, respectively; Table 5), however, note that estimates are on different scales. Estimates of genetic variation in trial A are based on PWD classes (Table 2), while in trial B estimates are based on amount of area debarked by pine weevils, and are not directly comparable. In both trials, estimates of heritability for plant height were high, with values above 0.4 (Table 5). The additive genetic correlation between PWD and height (*r_a_*) in Trial A was 0.184 (SE, 0.108), and not significantly different from 0 (*p* > 0.05). In Trial B, the correlation was 0.494 (SE, 0.138), and significantly different from 0 (*p* < 0.05).

**Table 5:** Additive genetic variance, error variance, heritability estimates and their SEs (std. error) for levels of pine weevil damage (PWD) in Norway spruce plants in Trials A and B.

Trait	Additive genetic variance (*V*_G_)	Error variance	Heritability (*h*^2^ = V_G_/V_T_)	*h*^2^ std. error
**Trial A**			
PWD class (0–5)	0.136	0.31	0.31	0.05
PWD mid-class value	0.006	0.01	0.34	0.05
Height (cm)	59.2	69.1	0.46	0.05
**Trial B**			
PWD (cm^2^)	0.02	2.0	0.1	0.04
Height (cm)	81.9	85.9	0.49	0.04


*In trial A, PWD was expressed in both discrete classes (0–5) and as a continuous variable (PWD mid-class, Table 2), while in trial B damage was expressed as stem area debarked (PWD, cm^*2*^).*

## Discussion

Presented results clearly showed that emblings were less frequently attacked by pine weevils, and when attacked, they received less damage than seedlings. Moreover, the observed variation in damage among half-sib families had a genetic component; we detected significant heritabilities for the levels of damage received, suggesting the potential to select plant material with greater pine weevil resistance. Further, there were minor differences in growth between emblings and seedlings, in accordance with patterns observed in previous studies of other conifers (e.g., Grossnickle and Major, 1994; O’Neill et al., 2005). We show here for the first time that, in addition to rapid propagation of superior genotypes, SE can be a valuable tool in efforts to counter major threats posed by insect pests and facilitate diverse other mechanistic investigations, as discussed below.

### Effects of Propagation Method on Plant Resistance

Propagation via SE clearly reduced PWD relative to propagation via seeds: in Trial A, emblings received less damage than seedlings, and in Trial B a lower proportion of emblings than seedlings were attacked. These results suggest that emblings and seedlings differ with respect to one or more plant traits affecting host plant choice and extent of bark-feeding by pine weevils. Factors that could potentially mediate such effects on herbivores are outlined below.

It is well-established that the *in vitro* environment associated with SE generates stress and increases secondary metabolite production in cultured tissues of various conifer species (Klimaszewska et al., 2016), and such effects can persist even when plants are grown from these tissues (Lamhamedi et al., 2000). Thus, Norway spruce emblings in our experiment could have had higher constitutive levels of chemical defenses than seedlings, in turn affecting traits relevant to herbivore host choice. For example, changes in the composition of volatiles emitted by plants are known to affects pine weevils’ orientation to potential food sources (Lundborg et al., 2016b). Thus, differences in chemical odors between emblings and seedlings could have affected the risk of plants being found or chosen by pine weevils. Likewise, the extent of bark-feeding was also reduced for emblings compared to seedlings, and higher levels of secondary metabolites could have reduced the emblings’ palatability. Similar reductions in pine weevil feeding have been observed for seedlings whose anti-herbivore defenses have been induced by treatment with the stress-related plant hormone methyl jasmonate (MeJA) (Fedderwitz et al., 2016; Lundborg et al., 2016a). Seedlings that have been exogenously treated with MeJA before exposure to pine weevils reportedly have higher levels of chemical defenses (phenolics and terpenoids), and suffer significantly less damage and mortality even 2 years after treatment (Zas et al., 2014). Hence, SE-generated changes in levels of chemical compounds relevant to plant resistance to pine weevils are likely to affect weevil feeding on emblings. Further studies are required to determine the underlying mechanisms and relevant defense traits mediating differences in anti-herbivore resistance between emblings and seedlings.

Both the SE process and herbivory are stressful for plants, and they induce responses involving similar signaling pathways (Wasternack and Hause, 2002). Thus, in addition to enhancing their secondary metabolite production, emblings’ early exposure to stress during SE could “prime” their biotic defenses, enabling them to respond rapidly to subsequent attacks. Accordingly, plants that have received signals that future attack is likely to occur or have previously been exposed to herbivore attack can deploy defenses more rapidly, and tend to be damaged less than “unprepared” counterparts (Karban and Baldwin, 1997; Kessler et al., 2006; Heil, 2009; Kim and Felton, 2013; Karban et al., 2014; Zhang et al., 2018). Moreover, genes with known importance in innate plant immunity are involved in plant embryogenesis. For instance, genes associated with the induction of Systemic Acquired Resistance (SAR) in plants are expressed early during SE in *Pinus radiata* (Aquea and Arce-Johnson, 2008). In addition, transgenic manipulations of Somatic Embryogenesis Receptor-like Kinase (*SERK*) expression have revealed direct links between *SERK* and both susceptibility to fungal pathogens and resistance to aphids in several plant species (Hu et al., 2005; Santos et al., 2009; Mantelin et al., 2011). Hence, early “priming” of biotic defenses could also be a relevant factor contributing to observed differences in levels of herbivore damage received by emblings and seedlings in our experiment.

It is important to note that we identified a previously unknown difference between emblings and seedlings with respect to plant resistance against insect herbivory, but are unable to determine the reasons for such a difference. However, this discovery in itself could provide a powerful new approach for elucidating the underlying plant defense mechanisms, for instance, through genetic and eco-physiological comparisons of emblings and seedlings. Improvements in such mechanistic understanding are greatly needed to address incomplete knowledge on the specific traits mediating plant defense (Erb, 2018), as well as developing sustainable solutions to current and future challenges posed by pests and various other stressors (Santamaria et al., 2013; Mitchell et al., 2016). Another important caveat is that we compared levels of insect herbivory between emblings and seedlings at only one time point, 2 years after planting. Our results could be considered in line with other studies showing that SE-mediated increases in secondary metabolite production have persisted for 2 years after planting (Fulzele and Satdive, 2003; Domínguez et al., 2010). However, whether greater secondary metabolite production leads to reduced levels of herbivore damage and confers a benefit to SE-plants, during a short span or an entire lifetime, remains to be investigated.

### Genetic Variation in Resistance Against PWD

We found significant additive genetic variation in plant resistance to PWD, but the extent of variation depended on the indicator of resistance examined. Heritability estimates for the levels of damage received by plants were significant for both measures of resistance, but heritability of damage expressed as classes was slightly greater than that expressed as amount of area debarked (Trial A vs. B, respectively; Table 5). However, due to differences in the scale of measurement, the magnitude of heritability estimates are not directly comparable. Furthermore, in trial B, more than half of the plants were undamaged, so the estimates also reflect the probability of plants being attacked. In Trial B, pine weevil pressure may have been too low to allow detection of large differences among families in resistance. Similarly, in a previous study of Norway spruce, genetic variation was observed in probability of attack, but not in amount of damage received on a site with low pine weevil pressure, while the opposite was found at another site with high damage incidence (Zas et al., 2017). We also detected differences in the genetic correlation between plant height and damage in the two trials; a significant positive correlation in Trial B but not Trial A. Again, this could be partly due to pine weevil host choice affecting the estimates more strongly in Trial B, where the incidence of damage was low, than in Trial A, where all plants were attacked (regardless of height growth).

Overall, our results indicate that traits contributing to the risk of attack and reducing the levels of PWD received by plants have a heritable component. Thus, future efforts to increase resistance to PWD through selection and breeding could potentially exploit this genetic variation. Furthermore, since the amount of PWD received by plants has a genetic basis, spruce families could also exhibit variation in their propensity or ability to develop SE-mediated defense mechanisms. Future studies will be required to examine genetic variation in stress responses associated with SE and if this variation is subsequently reflected in emblings’ susceptibility to pests.

## Conclusion

We present here the first evidence that propagation via SE may enhance plants’ resistance to insect herbivory, as shown by lower incidence of PWD in SE-propagated plants. Moreover, as we have discussed, there are sound theoretical grounds for expecting SE-mediated effects on defense to be a general feature and not specific to the spruce-pine weevil system. Thus, the findings indicate novel avenues to explore mechanisms underlying plant resistance, develop non-toxic measures against insect herbivores (and potentially other pests), elucidate various physiological processes, and formulate strategies to improve plants’ ability to cope with diverse stresses.

## Author Contributions

K-AH, NB, and GN conceived, designed and performed the experiments. K-AH analyzed the data and AP conducted the systematic review (Supplementary Information). AP wrote the manuscript with substantial inputs from K-AH, GN, and NB.

## Conflict of Interest Statement

The authors declare that the research was conducted in the absence of any commercial or financial relationships that could be construed as a potential conflict of interest.

## Supplementary Material

APPENDIX 1 Literature search examining whether or not previous studies have reported differences in susceptibility to pests between plants propagated via somatic embryogenesis and other non-tissue culturing methods (e.g., grown from seeds or cuttings).
