# Supplementary material for: Novel Avenues for Plant Protection: Plant Propagation by Somatic Embryogenesis Enhances Resistance to Insect Feeding
Source: Front Plant Sci. 2018 Oct 26;9:1553. doi: 10.3389/fpls.2018.01553 (PMC6217029; doi:10.3389/fpls.2018.01553)
Supplement: APPENDIX 1 — Literature search examining whether or not previous studies have reported differences in susceptibility to pests between plants propagated via somatic embryogenesis and other non-tissue culturing methods (e.g., grown from seeds or cuttings). [file Table_1.docx]

Supplementary Material

**Novel avenues for plant protection: Plant propagation by somatic embryogenesis enhances resistance to insect feeding**

Adriana Puentes*, Karl-Anders Högberg, Niklas Björklund, Göran Nordlander

*Correspondence: Corresponding Author: [adriana.puentes@slu.se](mailto:adriana.puentes@slu.se)

**1 Supplementary data – Appendix 1**

We conducted several comprehensive literature searches to determine whether previous studies have reported differences between plants propagated via somatic embryogenesis (SE) and other non-tissue culturing methods (e.g., grown from seeds or cuttings) with respect to herbivore damage/pest resistance and possible underlying mechanisms. The search methodology, specific questions addressed and results for each search are reported below.

**Search methodology**

We followed guidelines for systematic reviews presented by Khan *et al*. (2003) in our search and selection process, but did not perform a meta-analysis after selecting relevant papers. Instead, we summarized and qualitatively evaluated the evidence provided by the articles found. The process involves the following five steps:

- *Step 1: Framing questions for a review*

Problems to be addressed should be specified in the form of clear, unambiguous and structured questions before beginning the review work.

- *Step 2: Identifying relevant work*

The study selection criteria should flow directly from the review questions and be specified *a priori*. A first reading of article titles and abstracts should be conducted to efficiently remove spurious hits, and select relevant papers for the review.

- *Step 3: Assessing the quality of studies*

Selected studies (Step 2) should be subjected to a more refined quality assessment based on the full text. Papers passing this stage will serve as evidence for answering the questions.

- *Step 4: Summarizing the evidence*

Data synthesis consists of tabulation of each study’s characteristics, quality and effects as well as use of statistical methods if a meta-analysis is to be conducted.

- *Step 5: Interpreting the findings*

Any recommendations should be graded by reference to the strengths and weaknesses of the evidence.

(Reference: **Khan KS, Kunz R, Kleijnen J, Antes G. 2003.** Five steps to conducting a systematic review. *Journal of the Royal Society of Medicine* **96**: 118-121.)

All of our searches were conducted in Web of Knowledge (Databases: Web of Science Core Collection, Biosis Citation Index, CAB Abstracts and Global Health (CABI) and Scopus) using the “field = topic” search field, which searches for terms in the title, abstract or keywords. No time limit was applied, but language was restricted to English and document type was limited to Journal Article, Book Section, Serial, and Conference papers.

**Problems addressed and search results**

**Question 1.** *Do plants/seedlings produced through somatic embryogenesis receive different levels of herbivore damage compared to those not produced from tissue culturing?*

To find relevant articles to this question we conducted a search with the terms described in Table 1. Papers that qualified as relevant to the question had to have compared extent of herbivore damage/resistance to insects between SE-produced plants and counterparts that had not been produced through tissue culturing. The non-tissue-cultured group could include seed-germinated, vegetatively-propagated (e.g., from cuttings) or wild-growing plants in the lab or field. Studies examining transgenic plants (e.g., with *Bacillus Thuringiensis* genes inserted) micropropagated through somatic embryogenesis and subsequently tested in the lab/field for gene expression and levels of insect resistance (insect bioassays), were excluded. We also excluded papers that included propagated material originating from non-transgenic insect-resistant vs. susceptible families, as the effects of family and propagation method were confounded. We were specifically interested in finding studies that reported an effect of the SE process *per se* on plant resistance to insect herbivores.

In this search, we found no papers that met our criteria (Table 1). It yielded mostly papers on SE-propagated plant material that was genetically modified to express specific traits, and advantages of SE over other tissue culturing methods. However, when surveying abstracts, one paper caught our attention and led to subsequent searches:

**Beyene G, Chauhan RD, Wagaba H, Moll T, Alicai T, Miano D, Carrington JC, Taylor NJ. 2016.** Loss of *CMD2*-mediated resistance to cassava mosaic disease in plants regenerated through somatic embryogenesis. *Molecular Plant Pathology* **17**: 1095-1110.

This paper reports the loss of resistance to cassava mosaic disease in plant material genetically modified to express a specific and well-known disease resistance gene, after being propagated through SE. Given that herbivory and disease elicit similar stress responses in plants, we decided to expand our search beyond insect resistance and consider the potential relationship between the SE process and pathogen resistance.

**Question 2.** *Do embryos/plants produced through somatic embryogenesis exhibit different levels of pathogen resistance compared to those not produced from tissue culturing?*

To find relevant articles to this question we conducted another search with the terms described in Table 1. Papers that qualified as relevant to the question had to have compared extent of pathogen resistance between SE-produced plants and counterparts that had not been produced through tissue culturing.

This search yielded several papers that reported elimination of viruses/viroids from contaminated material following propagation via SE in different crops such as grapevines and citrus trees (Table 2). In studies reported in these papers, plant material known to be contaminated with a pathogen had been propagated via somatic embryogenesis, and subsequent examination of the plantlets produced through SE showed that they were pathogen-free. In some cases, SE-plants had been examined years later and continued to be virus-free. Thus, SE could be used as a tool to sanitize contaminated plant material, putatively due to a lack of vascular connection between somatic embryos and parental tissue, or inability of infected cells to embryonize (Peiró *et al*., 2005; Gambino *et al*., 2011). However, these papers do not directly address pathogen resistance *per se*, as sanitation does not involve any plant defence mechanisms or properties that deter pathogens from attacking or infecting a given cell. To compare degrees of pathogen resistance among different types of propagated tissue, exposure to pathogens under similar conditions is required and measures of inhibitory effects on the pathogen or levels of tissue infection is needed.

Even more interesting and relevant, our search also yielded papers describing defence reactions to fungal pathogens in SE tissue at different developmental stages. Production of secondary metabolites during all stages had reportedly reduced or inhibited fungal mycelial growth in agar cultures. Vookova *et al*. (2006) and Hrib *et al*. (2013) discuss the role of abscisic acid (ABA) and auxin in mediating these defence reactions, as they are known to be involved in plant responses to biotic stresses. Phenolic metabolism is promoted by ABA and given the high concentrations of ABA required in the SE process, SE tissues seem likely to produce high levels of phenolics, as manifested by browning during callus development. In references cited by Hrib *et al*. (2013), we also found a paper on browning of callus and insect resistance (Dowd *et al*., 1995; Table 2), which showed that older and browner callus (8-week-old) of *Zea mays* (corn) caused higher mortality of the corn earworm *Helicoverpa zea* than 5-week-old less brown callus. Overall, these papers suggest a connection between the SE process (e.g., growing conditions with high ABA levels) and the production of secondary metabolites, with potentially important consequences for plant defences against pathogens and insects.

It should be noted that an additional study confirmed that the SE treatment elicited a biotic defense response in the explant material used (Rutledge *et al*., 2013). However, the authors suggest that the defence response occurred at such high levels that it resulted in inhibition of the SE induction process, with the explant material remaining unresponsive to subsequent steps of the process and not developing into somatic embryos. The authors warn that only one genotype was examined and further studies on the potentially antagonistic relationship between defense responses and SE are required.

In line with the idea that the SE process and defense responses may be connected, the search also yielded a few papers on the role of genes activated during somatic embryogenesis (related to somatic embryogenesis receptor kinase, *SERK*, genes) and their roles in plant immunity (Table 2). These studies show that expression of *SERK* genes affects resistance to aphids and responses to fungal infections, suggesting that these genes are not only involved in growth and development but also in biotic and abiotic stress perception. Santos *et al*. (2009) specifically highlight the plant growth regulator auxin as a stress inducer during the process of somatic embryogenesis, in line with studies by Vookova *et al*. (2006) and Hrib *et al*. (2013) (Defence responses in somatic embryos, Table 2).

Given the results of this search on the increased production of secondary metabolites under the stressful conditions associated with the SE process, we conducted an additional search regarding the relationship between the SE process and changes in phytochemistry.

**Question 3.** *Do embryos/plants produced through somatic embryogenesis exhibit differences in the production of secondary metabolites compared to those not produced from tissue culturing?*

To find relevant articles to this question we conducted another search with the terms described in Table 1. Papers that qualified as relevant to the question had to have compared chemical profiles and/or levels of specific secondary compounds between SE-produced plants and counterparts that had not been produced through tissue culturing.

This search yielded several papers examining different properties of SE- vs ZE-produced plant material; in some cases phytochemistry was the main focus while in others it was just one of the properties examined (Table 3). These studies found that secondary metabolite levels were much higher in examined SE embryos/plants that had undergone somatic embryogenesis than in appropriate counterparts, in line with findings presented in the “Defence responses in somatic embryos” section (Question 2; Table 2). Most interestingly, we found several papers comparing ‘wild’ or non-tissue cultured mother plants to SE-derived plants showing that secondary metabolite levels continued to be elevated in emblings (not only in embryos), even 1-2 years after propagation (e.g., Lamhamedi *et al*., 2000; Fulzele & Sative, 2003). Accordingly, we also found papers showing that expression of genes involved in the biosynthesis of secondary metabolites was much higher in SE-derived material than in corresponding ZE-material. These results provide clear evidence that the process of somatic embryogenesis promotes production of secondary metabolites, and the resulting changes in phytochemistry early in embryo development can persist for several years.

As several of the papers identified in this search described studies of plants of medicinal/pharmaceutical interest, we conducted a fourth, specific search for papers concerning phytochemical differences in such plants.

**Question 4.** *Do medicinal plants/seedlings produced through somatic embryogenesis exhibit differences in the production of secondary metabolites compared to those not produced from tissue culturing?*

To find relevant articles to this question we conducted a final search with the terms described in Table 1. Papers that qualified as relevant to the question had to have compared chemical profiles and/or levels of specific secondary compounds between SE-produced medicinal plants and counterparts that had not been produced through tissue culturing.

This search yielded studies that confirmed that somatic embryogenesis can be exploited to increase yields of secondary metabolites, specifically products of medicinal plants (Table 2). The studies indicate that SE-derived plants often have higher levels of metabolites than field- or greenhouse-grown plants. However, production is not always increased following SE and results can vary depending on species and compounds of interest. Hayta *et al*. (2011) and Abdelsalam *et al*. (2017) found lower levels of measured secondary metabolites in SE vs. wild plants, while Wong *et al*. (2013) found similar levels. We also considered several reviews identified in the search to seek potential explanations for observed discrepancies in the effectiveness of SE. A few reviews of medicinal herbaceous and woody plants indicate that intensifying secondary metabolite production through tissue culturing depends on growth medium composition, physical factors and PGRs (plant growth regulators). Various PGRs and growth retardants may synergistically enhance metabolite production (Kulhari *et al*., 2012), but not all secondary metabolites are equally responsive. Hence, successful enhancement of secondary compound production via SE may depend on the plant species and metabolite of interest. Overall, these results confirm that phytochemical changes occur during the SE process itself (in line with previous search results), but show that increases in the production of secondary metabolites depend on the SE environment, plant species and metabolites considered.

**Summary and conclusion**

From the evidence gathered through all four searches, we conclude that the process of somatic embryogenesis induces responses that can result in genetic and chemical differences between emblings and plants generated via other non-tissue culturing methods. Such changes can have well-established effects on plant resistance to pathogens, and less well-established effects on resistance to insect herbivores, the latter based on two correlative studies concerning aphid resistance and a *SERK* gene (Mantelin *et al*., 2011) and the phenolic contents of calli and insect mortality (Dowd *et al*., 1995).The environment in which somatic embryogenesis occurs (i.e., the growth medium and physical conditions) appears to trigger stress responses that result in elevated expression of genes involved in biotic/abiotic defences. Overexpression of these genes appears to be one of the mechanisms that may increase production of protective secondary metabolites *in vitro*. Such responses to the SE process are commonly exploited to obtain metabolites of medicinal interest from plants that are endangered or difficult to extract. The positive effects of SE on secondary compound production may persist even when plants have established and are grown in field conditions. Overall, we conclude that our study provides the first direct evidence of a positive relationship between SE and plant resistance to insect herbivores*. Together with previous evidence of changes elicited by the SE process *per se*, it seems likely that the enhanced resistance to herbivory is mediated by stress responses triggered during SE that have been shown to subsequently affect resistance to pathogens.

***Note:** All of our systematic Web of Science searches (Table 1) were re-run on September 15^th^, 2018 and no new papers reporting a relationship between somatic embryogenesis and plant resistance to insect herbivory were found. Thus, the conclusion that our study provides the first direct evidence of a positive relationship between SE and plant resistance to insect herbivores remains supported.

**References**

**Abdelsalam A, Mahran E, Chowdhury K, Boroujerdi A, El-Bakry A. 2017.** NMR-based metabolomic analysis of wild, greenhouse, and in vitro regenerated shoots of *Cymbopogon schoenanthus* subsp. *proximus* with GC–MS assessment of proximadiol. *Physiology and Molecular Biology of Plants* **23**: 369-383.

**Beyene G, Chauhan RD, Wagaba H, Moll T, Alicai T, Miano D, Carrington JC, Taylor NJ. 2016.** Loss of *CMD2*-mediated resistance to cassava mosaic disease in plants regenerated through somatic embryogenesis. *Molecular Plant Pathology* **17**: 1095-1110.

**Bhattacharyya P, Kumaria S, Job N, Tandon P. 2015.** Phyto-molecular profiling and assessment of antioxidant activity within micropropagated plants of *Dendrobium thyrsiflorum*: a threatened, medicinal orchid. *Plant Cell, Tissue and Organ Culture* **122**: 535-550.

**Borroto-Fernandez EG, Sommerbauer T, Popowich E, Schartl A, Laimer M. 2009.** Somatic embryogenesis from anthers of the autochthonous *Vitis vinifera* cv. *domina* leads to arabis mosaic virus-free plants. *European Journal of Plant Pathology* **124**: 171-74.

**Domínguez F, Chávez M, Garduno-Ramirez ML, Chávez-Avila VM, Mata M, Cruz-Sosa F. 2010.** Honokiol and magnolol production by in vitro micropropagated plants of *Magnolia dealbata*, an endangered endemic Mexican species. *Natural Product Communications* **5**: 235-240.

**D’Onghia AM, Carimi F, Pasquale FD, Djelouah K, Martelli GP. 2001.** Elimination of citrus psorosis virus by somatic embryogenesis from stigma and style cultures. *Plant Pathology* **50**: 266-269.

**D'Onghia AM, Pasquale FD, Carimi F, Savino V, Crescimanno FG. 1997.** Somatic embryogenesis from style culture as a possible means for virus elimination in Citrus. *Journal of Phytopathology* **145**: 77-79.

**Dowd PF, Norton RA. 1995.** Browning – associated mechanisms of resistance to insects in corn callus tissue. *Journal of Chemical Ecology* **21**: 583-600.

**Filippini R, Caniato R, Vecchia FD, Cappelletti EM, Puricelli L, Piovan A, Innocenti G.** 2000. Somatic embryogenesis and indole alkaloid production in *Catharanthus roseus*. *Plant Biosystems* **134**: 179-184.

**Fulzele DP, Satdive RK. 2003.** Somatic embryogenesis, plant regeneration, and the evaluation of camptothecin content in *Nothapodytes foetida. In Vitro Cellular & Developmental Biology-Plant* **39**: 212-216.

**Gambino G, Bondaz J, Cuozzo D, Gribaudo I. 2007.** Virus eradication from grapevine somatic embryos. *Italus Hortus* **14**: 15-24.

**Gambino G, Bondaz J, Gribaudo I. 2006.** Detection and elimination of viruses in callus, somatic embryos and regenerated plantlets of grapevine. *European Journal of Plant Pathology* **114**: 397-404.

**Gambino G, Di Matteo D, Gribaudo I. 2009.** Elimination of grapevine fanleaf virus from three *Vitis vinifera* cultivars by somatic embryogenesis. *European Journal of Plant Pathology* **123**: 57-60.

**Gambino G, Navarro B, Vallania R, Gribaudo I, Di Serio F. 2011.** Somatic embryogenesis efficiently eliminates viroid infections from grapevines. *European Journal of Plant Pathology* **130**: 511-519.

**Gambino G, Vallania R, Gribaudo I. 2010.** In situ localization of grapevine fanleaf virus and phloem-restricted viruses in embryogenic callus of *Vitis vinifera*. *European Journal of Plant Pathology* **127**: 557-570.

**Ghaderi N, Jafari M. 2014.** Efficient plant regeneration, genetic fidelity and high-level accumulation of two pharmaceutical compounds in regenerated plants of *Valeriana officinalis* L. *South African Journal of Botany* **92**: 19-27.

**Goussard PG, Wiid J. 1991.** The use of somatic embryogenesis to eliminate leafroll-associated viruses in grapevines. *Deciduous Fruit Grower* **41**: 423-426.

**Gribaudo I, Cuozzo D, Ruffa P, Gambino G, Mannini F. 2009.** Virus eradication from grapevine minor cultivars at the IVV-CNR, Grugliasco Unit. *Italus Hortus* **16**: 194-197.

**Gribaudo I, Gambino G, Cuozzo D, Mannini F. 2006.** Attempts to eliminate grapevine rutestris stem pitting-associated virus from grapevine clones. *Journal of Plant Pathology* **88**: 293-298.

**Hayta S, Gurel A, Akgun I, Altan F, Ganzera M, Tanyolac B, Bedir E. 2011.** Induction of *Gentiana cruciata* hairy roots and their secondary metabolites. *Biologia* **66**: 618-625.

**Heese A, Hann DR, Gimenez-Ibanez S, Jones AME, He K, Li J, Schroeder JI, Peck SC, Rathjen JP. 2007.** The receptor-like kinase *SERK3/BAK1* is a central regulator of innate immunity in plants. *Proceedings of the National Academy of Sciences of the United States of America* **104**: 12217-12222.

**Hrib J, Adamec V, Sedlacek V, Vookova B. 2013.** Passive defense in somatic embryos of *Abies alba* from two cell lines tested with *Phaeolus schweinitzii*. *Dendrobiology* **70**: 83-91.

**Hu H, Xiong L, Yang Y. 2005.** Rice *SERK1* gene positively regulates somatic embryogenesis of cultured cell and host defense response against fungal infection. *Planta* **222**: 107-117.

**Isah T, Mujib A. 2015.** In vitro propagation and camptothecin production in *Nothapodytes nimmoniana*. *Plant Cell, Tissue and Organ Culture* **121**: 1-10.

**Iyer RI, Jayaraman G, Gopinath PM, Sita GL. 2000.** Direct somatic embryogenesis in zygotic embryos of nutmeg (*Myristica fragrans* Houtt.). *Tropical Agriculture* **77**: 98-105.

**Kulhari A, Sheorayan A, Kalia S, Chaudhury A, Kalia RK. 2012.** Problems, progress and future prospects of improvement of *Commiphora wightii* (Arn.) Bhandari, an endangered herbal magic, through modern biotechnological tools: a review. *Genetic Resources and Crop Evolution* **59**: 1223-1254.

**Lamhamedi MS, Chamberland H, Bernier PY, Tremblay FM. 2000.** Clonal variation in morphology, growth, physiology, anatomy and ultrastructure of container-grown white spruce somatic plants. *Tree Physiology* **20**: 869-880.

**Mantelin S, Peng HC, Li B, Atamian HS, Takken FL, Kaloshian I. 2011.** The receptor-like kinase *SlSERK1* is required for *Mi-1*-mediated resistance to potato aphids in tomato. *The Plant Journal* **67**: 459-471.

**Máthé Á. 2015.** *Medicinal and Aromatic Plants of the World*. Dordrecht, Netherlands: Springer.

**Maximova SN, Florez S, Shen X, Niemenak N, Zhang Y, Curtis W, Guiltinan MJ. 2014.** Genome-wide analysis reveals divergent patterns of gene expression during zygotic and somatic embryo maturation of *Theobroma cacao* L., the chocolate tree. *BMC Plant Biology* **14**: 185.

**Meziane M, Frasheri D, Carra A, Boudjeniba M, D’Onghia AM, Mercati F, Djelouah K, Carimi F. 2017.** Attempts to eradicate graft-transmissible infections through somatic embryogenesis in *Citrus* ssp. and analysis of genetic stability of regenerated plants. *European Journal of Plant Pathology* **148**: 85-95.

**Mohamed ESA, Gomaa A, Danial N. 2014.** In vitro regeneration and somatic embryogenesis in *Citrus*. *Journal of Plant Tissue Culture and Biotechnology* **24**: 247-262.

**Peiró R, Gammoudi N, Yuste A, Olmos A, Gisbert C. 2015.** Mature seeds for in vitro sanitation of the grapevine leafroll associated virus (*GLRaV-1* and *GLRaV-3*) from grape (*Vitis vinifera* L.). *Spanish Journal of Agricultural Research* **13**: e1005

**Pinto G, Loureiro J, Lopes T, Santos C. 2004.** Analysis of the genetic stability of *Eucalyptus globulus* Labill. somatic embryos by flow cytometry. *Theoretical and Applied Genetics* **109**: 580-587.

**Quainoo AK, Wetten AC, Allainguillaume J. 2008.** The effectiveness of somatic embryogenesis in eliminating the cocoa swollen shoot virus from infected cocoa trees. *Journal of Virological Methods* **149**: 91–96.

**Rutledge RG, Stewart D, Caron S, Overton C, Boyle B, MacKay J, Klimaszewska K. 2013.** Potential link between biotic defense activation and recalcitrance to induction of somatic embryogenesis in shoot primordia from adult trees of white spruce (*Picea glauca*). *BMC Plant Biology* **13**: 116.

**Santos MO, Romano E, Vieira LS, Baldoni AB, Aragao FJL. 2011.** Suppression of *SERK* gene expression affects fungus tolerance and somatic embryogenesis in transgenic lettuce. *Plant Biology* **11**: 83-89.

**Shinde AN, Malpathak N, Fulzele DP. 2010.** Determination of isoflavone content and antioxidant activity in *Psoralea corylifolia* L. callus cultures. *Food Chemistry* **118**: 128-132.

**Sridevi V, Giridhar P, Ravishankar GA. 2010.** Free diterpenes cafestol and kahweol in beans and in vitro cultures of *Coffea* species. *Current Science* **99**: 1101-1104.

**Szypuła W, Pietrosiuk A, Suchocki P, Olszowska O, Furmanowa M, Kazimierska O. 2005.** Somatic embryogenesis and in vitro culture of *Huperzia selago* shoots as a potential source of huperzine A. *Plant Science* **168**: 1443-1452.

**Tao L, Zhao Y, Wu Y, Wang Q, Yuan H, Zhao L, Wengdong G, You X. 2016.** Transcriptome profiling and digital gene expression by deep sequencing in early somatic embryogenesis of endangered medicinal *Eleutherococcus senticosus* Maxim. *Gene* **578**: 17-24.

**Von Aderkas P, Teyssier C, Charpentier JP, Gutmann M, Pâques L, Le Metté C, Ader K, Label P, Kong L, Lelu-Walter MA. 2015.** Effect of light conditions on anatomical and biochemical aspects of somatic and zygotic embryos of hybrid larch (*Larix× marschlinsii*). *Annals of Botany* **115**: 605-615.

**Vookova B, Hřib J, Adamec V. 2006.** Defence reactions of developing somatic embryos of Algerian fir (*Abies numidica*). *Forest Pathology* **36**: 215-224.

**Yeoman MM, Yeoman CL. 1996.** Manipulating secondary metabolism in cultured plant cells. *New Phytologist* **134**: 553-569.

**2 Supplementary figures and tables**

**Table 1.** Search terms, date each search was conducted, number of papers obtained (hits) after removing duplicates, and number of relevant papers (including papers added manually after the search was conducted) for each of the questions (1-4) addressed in a systematic search for studies comparing indicated properties of SE-generated vs. non-tissue cultured plant material.

***Note:** All of our systematic Web of Science searches were re-run on September 15^th^, 2018 and no new papers reporting a relationship between somatic embryogenesis and plant resistance to insect herbivory were found. Thus, the conclusion that our study provides the first direct evidence of a positive relationship between SE and plant resistance to insect herbivores remains supported.

| Question | Search terms | Date conducted | Hits (after deduplication) | Relevant to question |
| --- | --- | --- | --- | --- |
| 1 | ((“somatic embryo*” OR embling* or “SE plants”) AND (herbivor* or insect*)) | May 14^th^, 2017 | 183 | 0 |
| 2 | ((“somatic embryo*” OR embling*) AND (“pathogen”)) | July 24^th^, 2017 | 362 | 27 |
| 3 | ((“somatic embryo*” OR “embling*”) AND (“zygotic”)) AND ((“secondary compound*” OR “secondary metabolite*”) OR (“tannin*” OR “phenolic*”) OR (“phytochem*”)) | July 28^th^, 2017 | 48 | 8 |
| 4 | ((“somatic embryo*” OR “embling*”) AND (“zygotic” OR “wild” OR “mother plant*” OR “clon*”) AND (“medicin*”) AND (“secondary compound*” OR “metabolite*” OR “chemi*” OR “phenol*”))) | July 28^th^, 2017 | 30 | 11 |

**Table 2.** List of relevant papers found for question 2 in the systematic search presented per topic, together with the most relevant findings per topic group. Papers in brackets were not found in the Web of Science search but were found to be relevant and added manually to the results.

| Topic | Papers and focal crops | Main findings |
| --- | --- | --- |
| Sanitation (disease elimination) and somatic embryogenesis | **Grapevine**  Goussard *et al*. (1991)  Gribaudo *et al*. (2006)  Gambino *et al*. (2006)  Gambino *et al*. (2007)  Borroto-Fernandez *et al*. (2009)  Gambino *et al*. (2009)  Gribaudo *et al*. (2009)  Gambino *et al*. (2010)  Gambino *et al*. (2011)  Peiró *et al*. (2015)  **Citrus trees**  D’Onghia *et al*. (1997)  D’Onghia *et al*. (2001)  Mohamed *et al*. (2014)  Meziane *et al*. (2017)  **Cocoa trees**  Quainoo *et al*. (2008)  **Avocado trees**  Suarez *et al*. (2005)  **Cassava**  Beyene *et al*. 2016 | All these studies found that pathogen-contaminated material (in indicated crops) was disease-free after propagation via SE, except the studies on avocado (Suarez *et al*., 2005), in which sanitation was not successful, and cassava, in which transgenic material lost its resistance to a virus (Beyene *et al*., 2016). However, these papers do not directly address pathogen resistance, as sanitation does not involve plant defence mechanisms or properties that deter pathogens from attacking a given cell. |
| Defence responses of somatic embryos/tissue | [Dowd *et al*. (1995)]  [Terho *et al*. (2000)]  Vookova *et al*. (2006)  Hrib *et al*. (2013)  Rutledge *et al*. (2013)  [Nawrot-Chorabik *et al*. (2016)] | Vookova *et al*. (2006) and Hrib *et al*. (2013) examined somatic embryos’ defence responses to pathogens. They found that release of secondary metabolites by somatic tissue in different stages reduces/inhibits mycelial growth of a fungus. High concentrations (*inter alia*) of abscisic acid (ABA) and auxin are required during the SE process, and promote formation of phenolic compounds. Thus, they could mediate the observed defence responses. Phenolic compounds are manifested as callus browning. In references cited by Hrib *et al*. (2013) we found a paper on callus browning in corn (Dowd *et al*., 1995) showing that older, browner callus caused significantly higher mortality in the corn earworm *Helicoverpa zea* than younger less brown callus. These studies suggest a relationship between the SE process *per se* and secondary metabolism, with profound potential implications for plant defense reactions.  We also included a study referred to by Vookova *et al*. (2006), presented by Terho *et al*. (2000), and other papers citing it. One, by Nawrot-Chorabik *et al*. (2016) was included in the relevant results. The two additional papers describe investigations of defense responses to fungal pathogens in different types of coniferous embryogenic tissue.  Another study (Rutledge *et al*., 2013) confirms that SE treatment can elicit enhanced biotic defence responses at the molecular level. However, the observed response was so strong that it subsequently inhibited the SE process, so the explant material did not mature into somatic embryos. Only one genotype was examined and further studies on the potentially antagonistic relationship between defense responses and SE are required. |
| Somatic embryogenesis genes and their role in plant immunity | Hu *et al*. (2005)  Heese *et al*. (2007)  Santos *et al*. (2009)  Mantelin *et al*. (2011) | These studies show that Somatic Embryogenesis Receptor Kinase (*SERK*) genes, which are activated during somatic embryogenesis, play important roles in host defence against pathogens and resistance to insect pests. |

**Table 3.** List of relevant papers found for questions 3 and 4 in the systematic search presented per topic, together with the most relevant findings per topic group. Papers in brackets were not found in the Web of Science search but were found to be relevant and added manually to the results.

| Topic | Papers | Main findings |
| --- | --- | --- |
| Differences between somatic and zygotic embryos in secondary metabolite composition | [Yeoman & Yeoman (1996)]  Lamhamedi *et al*. (2000)  Iyer *et al*. (2000)  Pinto *et al*. (2004)  Fulzele & Satdive (2003)  Szypula *et al*. (2005)  [Sridiva *et al*. (2010)]  von Aderkas *et al*. (2015) | These papers show that secondary metabolite levels were much higher in plants that had undergone somatic embryogenesis than in zygotic counterparts (Sridiva *et al*., 2010 was not found in the search, but found to be relevant and added to the results). Lamhamedi *et al*. (2000), Pinto *et al*. (2004) and von Aderkas *et al*. (2015) also compared other properties of SE and ZE plants (e.g., anatomy and morphology). Iyer *et al*. (2000), cite a study (Yeoman & Yeoman, 1996) indicating that cultured plant cells (not specifically SE) can be used to produce secondary compounds of commercial interest. |
| Expression of genes involved in biosynthesis of secondary metabolites | Maximova *et al*. (2014)  Tao *et al*. (2016) | These studies detected enhanced expression of genes involved in the biosynthesis of secondary metabolites (e.g. flavonoids, Maximova *et al*., 2014) during the SE process. Results suggest that the developing tissues may be stressed during SE maturation. |
| Question 4. Differences in phytochemistry between SE and non-tissue cultured medicinal plants | **Comparison with field or greenhouse grown plants**  Filipinni *et al*. (2000)  Domínguez *et al*. (2010)  [Shinde *et al*. (2010)]  Wong *et al*. 2013  Ghaderi & Jafari (2014)  Bhattacharyya *et al*. (2015)  Hayta *et al*. (2011)  Abdelsalam *et al*. (2017)  **Reviews**  Kulhari *et al*. (2012)  Isah and Mujib (2015)  Mathe *et al*. (2015) | These papers show that SE can be exploited to increase yields of secondary metabolites, specifically products of medicinal plants. SE-derived plants often have higher levels of metabolites than field- or greenhouse-grown plants. However, this can vary with species and compound of interest. Hayta *et al*. (2011) and Abdelsalam *et al*. (2017) found lower levels of measured secondary metabolites in SE vs. wild plants, while Wong *et al*. (2013) found similar levels. A few reviews of medicinal herbaceous and woody plants indicate that increases in secondary metabolite production through tissue culturing depend on growth medium composition, physical factors and PGRs. Hence, successful enhancement of secondary compound production may depend on the plant species and metabolites considered. |
